# Upregulation of IGF-1R Expression during Neoadjuvant Therapy Predicts Poor Outcome in Breast Cancer Patients

**DOI:** 10.1371/journal.pone.0117745

**Published:** 2015-02-13

**Authors:** Sandra Heskamp, Otto C. Boerman, Janneke D. M. Molkenboer-Kuenen, Carla A. Wauters, Luc J. A. Strobbe, Caroline M. P. W. Mandigers, Peter Bult, Wim J. G. Oyen, Winette T. A. van der Graaf, Hanneke W. M. van Laarhoven

**Affiliations:** 1 Department of Nuclear Medicine, Radboud University Medical Center, Nijmegen, The Netherlands; 2 Department of Medical Oncology, Radboud University Medical Center, Nijmegen, The Netherlands; 3 Department of Pathology, Canisius-Wilhelmina Hospital, Nijmegen, The Netherlands; 4 Department of Surgery, Canisius-Wilhelmina Hospital, Nijmegen, The Netherlands; 5 Department of Internal Medicine, Canisius-Wilhelmina Hospital, Nijmegen, The Netherlands; 6 Department of Pathology, Radboud University Nijmegen Medical Centre, Nijmegen, The Netherlands; 7 Department of Medical Oncology, Academic Medical Center, University of Amsterdam, Amsterdam, the Netherlands; Institute of Biomedicine, FINLAND

## Abstract

**Introduction:**

The insulin-like growth factor 1 receptor (IGF-1R) may be involved in the development of resistance against conventional cancer treatment. The aim of this study was to assess whether IGF-1R expression of breast tumors changes during neoadjuvant therapy and to study whether these changes were associated with survival.

**Methods:**

Paraffin embedded tumor tissue was collected from pretreatment biopsies and surgical resections of 62 breast cancer patients who were treated with neoadjuvant chemotherapy or endocrine therapy. IGF-1R expression was determined immunohistochemically and compared before and after treatment.

**Results:**

High membranous IGF-1R expression at diagnosis correlated significantly with ER positivity, low tumor stage (stage I/II) and longer overall survival (p < 0.05). After neoadjuvant treatment, membranous IGF-1R expression remained the same in 41 (65%) tumors, was upregulated in 11 (18%) tumors and downregulated in 11 (18%) tumors. Changes in membranous IGF-1R expression were associated with overall survival (log-rank test: p = 0.013, multivariate cox-regression: p = 0.086). Mean overall survival time for upregulation, no change, and downregulation in IGF-1R expression was 3.0 ± 0.5 years, 7.3 ± 1.0 years and 15.0 ± 1.8 years, respectively. Changes in other parameters were not significantly associated with survival.

**Conclusion:**

Neoadjuvant therapy can induce changes in IGF-1R expression. Upregulation of IGF-1R expression after neoadjuvant treatment is a poor prognostic factor in breast cancer patients, providing a rationale for incorporating anti-IGF-1R drugs in the management of these patients.

## Introduction

The insulin-like growth factor 1 receptor (IGF-1R) is a receptor tyrosine kinase that plays a role in cancer development and progression.[1–3] In breast cancer, its expression is positively correlated with the presence of the estrogen receptor (ER).[4,5] Approximately 40 to 60% of ER-positive tumors express IGF-1R, while expression in ER-negative tumors is only 10 to 20%.[6] In general, IGF-1R correlates with good prognostic markers, such as ER positivity, older age, lower grade and human epidermal growth factor receptor 2 (HER2)-negativity. However, its expression has differential effects in the different breast cancer subtypes. For example, IGF-1R expression has been shown to be positively correlated with improved breast cancer specific survival among patients with ER positive tumors, while its expression was associated with an inferior prognosis in patients with HER2-overexpressing or triple negative tumors.[6–9]

IGF-1R expression can change during breast cancer treatment. Preclinical studies have shown that IGF-1R expression can be upregulated by estrogen and downregulated by tamoxifen.[10–13] Moreover, a clinical study in patients who received adjuvant treatment with tamoxifen showed a decrease in IGF-1R expression in tamoxifen-resistant recurrences compared to the primary tumor.[14] IGF-1R may also play a role in resistance to several types of treatment. For example, hyperactivation of IGF-1R has been shown to be involved in cisplatin resistance of ovarian cancer cells, while in breast and colorectal cancer cells, IGF-1R has been associated with resistance to 5-fluorouracil (5-FU).[15–17] Moreover, cross-talk between HER2 and IGF-1R by the formation of heterodimers can contribute to trastuzumab resistance.[18–20]

In summary, IGF-1R has a prognostic value in breast cancer, its expression can change during treatment, and it may play a role in resistance to conventional breast cancer therapies.

In the present study we investigated the dynamics of IGF-1R expression during neoadjuvant breast cancer treatment, in order to determine whether the expression of IGF-1R in human breast tumors can be affected by neoadjuvant breast cancer treatment and whether changes are associated with survival.

## Material and Methods

### Patients and tumor tissue

Breast cancer patients who received neoadjuvant treatment between 1989 and 2010 in the Radboud university medical center or Canisius-Wilhelmina Hospital in Nijmegen were selected from the population-based cancer registry. All patients from whom paraffin embedded material was available from time of diagnosis (biopsy of primary tumor) and at surgical resection of the primary tumor, were included in the study. Patients from whom only a fine needle aspiration or material from another site than the primary tumor available was, were excluded. Also, patients with a complete pathological response were excluded, since no material was available to analyze post treatment IGF-1R expression. In total, paraffin embedded material was available from 62 patients. These patients received neoadjuvant chemotherapy or endocrine therapy. Chemotherapy consisted of 1) cyclophosphamide, methotrexate, fluorouracil (CMF), 2) an anthracycline based schedule or 3) a taxane-based schedule. Endocrine therapy consisted of tamoxifen, anastrozole or letrozole. Patients received 2 to 8 cycles of chemotherapy. The average time between biopsy and surgery was 5.2 ± 0.2 and 5.6 ± 0.7 months for patients who received chemotherapy or endocrine therapy, respectively.

The expression of IGF-1R, ER, PR, HER2, Ki67 and cleaved caspase-3 was analyzed immunohistochemically. Additional information on neoadjuvant therapy, pathological parameters, clinical parameters, and survival data were collected from patient files. This retrospective study has been approved by the regional ethics review committee (CMO Regio Arnhem-Nijmegen) and the need for written informed consent from the patient was waived by the ethics committee. All the data in this study was anonymized.

### Immunohistochemistry

Tumor sections (4 μm) were stained for IGF-1R (3027, Cell Signaling), ER (RM-9101-S1, Neomarkers), PR (C89F1, Cell Signaling) and HER2 (A0485, Dako). First, endogenous peroxidase activity was blocked with 3% H_2_O_2_ in PBS (10 minutes at room temperature (RT) and antigen retrieval was performed in 10 mM sodiumcitrate, pH 6.0 for 10 minutes at 99°C. Subsequently, for ER, PR and HER2, nonspecific binding was blocked by incubation with 20% normal goat serum (30 minutes at RT). Tumor sections were incubated with the primary antibodies for 1–2 h at RT (HER2) or overnight at 4°C (ER, PR and IGF-1R). ER, PR and HER2 staining was followed by incubation with a goat-anti-rabbit biotinylated secondary antibody (Vector Laboratories, Burlingame, CA) for 30 min at RT and avidin-biotin-enzyme complex (Vector Laboratories, Burlingame, CA) for 30 min at RT. IGF-1R immunostaining was followed by incubation with Poly-HRP-Anti-mouse/rabbit/rat IgG (DPVO110HRP, Immunologic, Duiven, The Netherlands) for 30 minutes at RT. Finally, 3, 3'-diaminobenzidine (DAB) was used to develop the tumor sections. Membranous IGF-1R and HER2 expression was scored as negative (0), incomplete weak (1+), complete weak to moderate (2+) and strong (3+) membrane staining (Table 1). Tumors with a HER2 score of 3+ were considered HER2 positive. HER2 expression was only analyzed by immunohistochemistry, since no frozen tissue was available for FISH. IGF-1R cytoplasmic staining was scored as negative (0), weakly positive (1+) or strongly positive (2+). The percentage ER and PR positive cells was scored using an eye-piece grid. A tumor was considered positive if more than 1% of the tumor cells stained positive. The staining was scored by two independent readers who were blinded to the type of treatment and clinical outcome.

**Table 1:** Scoring of membranous IGF-1R expression.

Score	Staining pattern
0	No staining is observed, or membrane staining is observed in <10% of the tumor cells
1+	A faint/barely perceptible membrane staining is detected in >10% of tumor cells. The cells exhibit incomplete membrane staining
2+	A weak to moderate complete membrane staining is observed in >10% of tumor cells
3+	A strong complete membrane staining is observed in >10% of tumor cells

Cleaved caspase-3 (9661, Cell Signaling) and Ki67 (RM-9106S1, Thermo Scientific) staining was performed for all patients from the Radboud University Nijmegen Medical Centre (n = 42). In short, endogenous peroxidase activity was blocked and antigen retrieval was performed as described previously. Non-specific binding was blocked by incubation with 20% NGS (caspase-3) or 20% normal swine serum (Ki67), followed by incubation with the primary antibodies. Slides were incubated with a secondary peroxidase labeled swine-anti-rabbit antibody (Ki67) or a goat-anti-rabbit biotinylated secondary antibody followed by an avidin-biotin-enzyme complex (Caspase-3). DAB was used to develop the tumor sections. The percentage of positive tumors cells was estimated using an eye-piece grid.

### Statistical analysis

Statistical analyses were performed using IBM Statistics SPSS v20. Categorical variables were presented as frequencies and continuous variables as mean ± standard deviation (sd). To test whether IGF-1R expression correlated with clinical, pathological or other immunohistochemical parameters, pearson chi-square tests were performed for categorical variables. Mann-Whitney tests (2-group comparisons) or Kruskall-Wallis tests (more than 2 group comparisons) were performed for continuous variables.

Correlations between clinical, pathological, and immunohistochemical variables and overall survival (OS) were analyzed by Kaplan-Meier survival test, and differences were calculated with the log-rank test. OS was defined as the time from diagnosis (date of biopsy) until the date of death or last follow-up for patients alive. Survival plots were estimated by the Kaplan-Meier method. Survival analyses for continuous variables and multivariate survival analyses were performed by using Cox proportional hazard regression models. All tests were two-sided and a p < 0.05 was considered significant.

## Results

### Patient characteristics

IGF-1R expression of biopsy and surgical resection material was analyzed for all 62 patients. One patient was diagnosed with two primary tumors which were analyzed separately. Therefore, the total number of tumors analyzed was 63. Patient and tumor characteristics are summarized in Table 2. Nine patients (15.9%) presented with metastatic disease at time of diagnoses. All patients were female and mean age at diagnosis was 55 ± 13 years. For 42 out of 62 patients (68%), menopausal status was reported in the patients files. In total, 20 (48%) of these patients were premenopausal and 22 (52%) were post menopausal.

**Table 2:** Patient (N = 62) and tumor (N = 63) characteristics.

	N (%)
**Age at diagnosis (mean ± sd)**	55 ± 13
**Tumor morphology**	
Ductal carcinoma	45 (71%)
Lobular carcinoma	10 (16%)
Ductal + lobular carcinoma	4 (6%)
Other	4 (6%)
**Stage**	
Stage I	1 (2%)
Stage II	13 (21%)
Stage III	37 (60%)
Stage IV	9 (15%)
Unknown	2 (3%)
**Neoadjuvant chemotherapy**	49 (79%)
CMF	7 (11%)
Anthracycline-based	13 (21%)
Taxane-based	29 (48%)
**Neoadjuvant anti-estrogen therapy**	13 (21%)
Anastrozole	5 (8%)
Letrozole	3 (5%)
Tamoxifen	5 (8%)

Patients who received neoadjuvant chemotherapy (n = 49) were treated with CMF (n = 7), an anthracycline based schedule (n = 13) or a taxane-based schedule (n = 29). Patients who received anti-estrogen treatment were treated with anastrozole (n = 5), letrozole (n = 3) or tamoxifen (n = 5). Immunohistochemical analysis showed that 79% of all patients were diagnosed with an ER positive tumor, 51% with a PR positive tumor, 43% with a HER2 positive tumor, and 6% with a triple negative tumor (ER, PR and HER2 negative).

### IGF-1R expression

**Fig 1:**
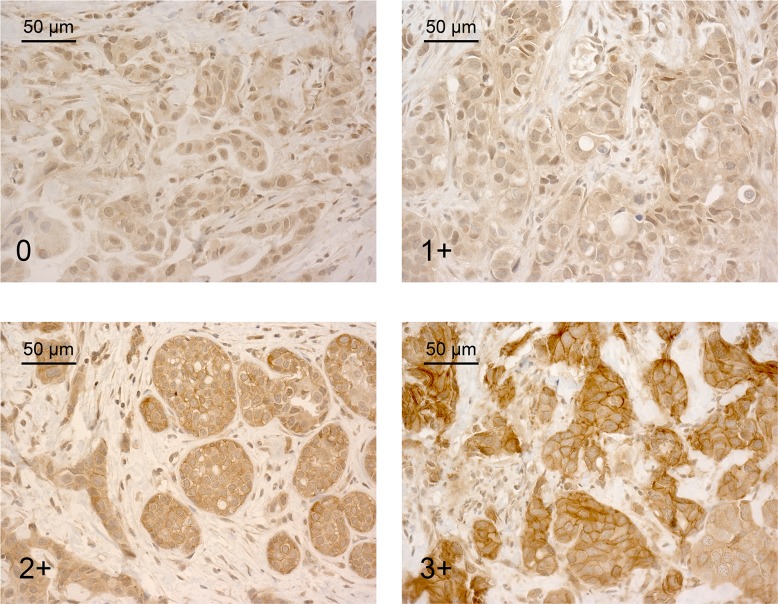
Examples of IGF-1R immunostaining of breast tumors. Scoring of IGF-1R membrane expression is depicted in the lower left corner. Magnification 400X, DAB staining.

Table 3 summarizes the results of the immunohistochemical analyses. At time of diagnosis, IGF-1R membrane expression was scored 0, 1+, 2+, and 3+ for 12 (19%), 18 (29%), 26 (41%), and 7 (11%) tumors, respectively. Examples of IGF-1R membrane staining are presented in Fig. 1. Membranous IGF-1R expression correlated significantly with ER and PR expression at time of diagnosis (χ^2^-test, p = 0.005 and 0.001). Moreover, it was significantly associated with tumor stage (χ^2^-test, p = 0.011). Patients with tumors with low or absent membranous IGF-1R expression (0 or 1+) presented more frequently with locally advanced or metastatic disease. Membranous IGF-1R expression at time of diagnosis did not differ significantly between chemotherapy or endocrine treated patients and was not correlated with any of the other clinical, pathological and immunohistochemical parameters. Cytoplasmic IGF-1R expression at time of diagnosis was scored as 1+ and 2+ for 60 (95%) and 3 (5%) tumors, respectively. Cytoplasmic staining did not correlate with other immunohistochemical, pathological or clinical parameters.

**Table 3:** Immunohistochemical markers of the 63 breast tumors.

	Pretreatment	Posttreatment
**ER**		
Positive	50 (79%)	54 (86%)
Negative	12 (19%)	8 (13%)
Unknown	1 (2%)	1 (2%)
**PR**		
Positive	32 (51%)	26 (41%)
Negative	29 (46%)	37 (59%)
Unknown	2 (3%)	0 (0%)
**HER2**		
Positive	27 (43%)	22 (35%)
Negative	30 (48%)	41 (65%)
Unknown	6 (10%)	0 (0%)
**IGF-1R membrane**		
0	12 (19%)	6 (10%)
1+	18 (29%)	30 (48%)
2+	26 (41%)	20 (32%)
3+	7 (11%)	7 (11%)
**IGF-1R cytoplasm**		
0	0 (0%)	2 (3%)
1+	60 (95%)	61 (97%)
2+	3 (5%)	0 (0%)

To determine whether IGF-1R expression changed during neoadjuvant treatment, expression at diagnosis was compared to expression at time of surgery. Membranous IGF-1R expression remained the same in 41 (65%) tumors, was upregulated in 11 (18%) tumors and downregulated in 11 (18%) tumors. The number of tumors showing an increase or decrease in membranous IGF-1R expression did not differ significantly between endocrine treated or chemotherapy treated patients. However, IGF-1R showed a trend towards more downregulation in the endocrine-treated group; IGF-1R was downregulated in 4/13 tumors and upregulated in 1/13 tumors. For patients who received chemotherapy, IGF-1R was downregulated in 7/50 tumors and upregulated in 10/50 tumors. Upregulation of membranous IGF-1R was significantly associated with high tumor stage at diagnosis (χ^2^-test, p = 0.04). Changes in membranous IGF-1R expression did not correlate with other clinical, pathological or immunohistochemical variables. Changes in cytoplasmic IGF-1R expression occurred less frequently. In 5 tumors (8%), a downregulation was observed, while none of the tumors showed an upregulation. Changes in cytoplasmic IGF-1R expression were not associated with any of the other immunohistochemical, pathological or clinical parameters.

### Survival analysis

**Fig 2:**
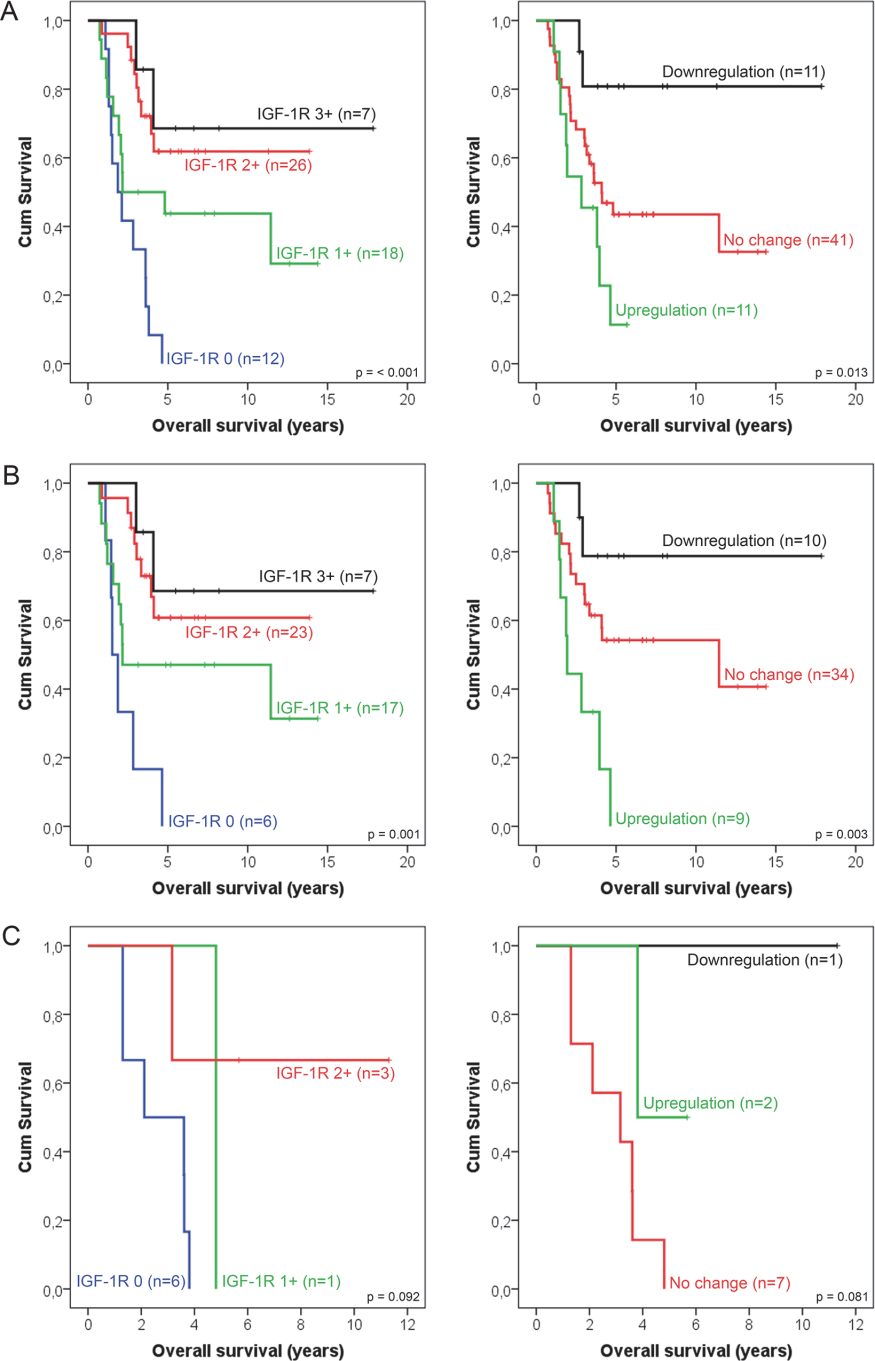
Kaplan-Meier survival curves. Kaplan-Meier survival curve estimates showing the effect of baseline IGF-1R expression (left) and changes in IGF-1R expression (right) on overall survival in the total population (**A**), and subgroup analysis in patients with non-metastatic disease (**B**) and metastatic disease (**C**).

Mean follow-up of patients included in the study was 4.6 ± 0.4 years (range 0.7–17.9 years). Mean OS of patients with stage I-III disease was 9.5 ± 1.2 years, compared with 4.6 ± 1.1 years for patients with stage IV disease (log-rank test, p = 0.17). Membranous IGF-1R expression at diagnosis was significantly associated with OS (log-rank test, p < 0.001, Fig. 2A). Mean OS time for patients with 0, 1+, 2+ and 3+ tumors was 2.4 ± 0.4 years, 6.9 ± 1.4 years, 9.7 ± 1.1 years, and 13.4 ± 2.6 years, respectively. Subgroup analysis for patients with metastatic versus non-metastatic disease is presented in Fig. 2B and 2C. Since membranous IGF-1R expression strongly correlated with ER expression and tumor stage, multivariate Cox regression analysis was performed to correct for these variables. In multivariate analysis, the correlation between membranous IGF-1R expression and OS remained significant (Table 4). Membranous and cytoplasmic IGF-1R expression after neoadjuvant treatment were not significantly associated with OS.

**Table 4:** Cox proportional hazard regression analysis.

	Univariate		Multivariate	
	HR (95%CI)	p	HR (95% CI)	p
**Baseline IGF-1R expression**		<0.001		0.015
1+ vs 0	0.38 (0.16–0.89)	0.026	0.36 (0.15–0.88)	0.025
2+ vs 0	0.17 (0.07–0.42)	<0.001	0.24 (0.09–0.62)	0.003
3+ vs 0	0.12 (0.03–0.55)	0.006	0.21 (0.04–1.04)	0.056
**ER expression**				
positive vs negative	0.42 (0.20–0.92)	0.029	0.53 (0.22–1.25)	0.15
**Tumor stage**				
III/IV vs I/II	3.52 (1.07–11.6)	0.038	2.26 (0.62–8.13)	0.21
	**Univariate**		**Multivariate**	
	**HR (95%CI)**	**p**	**HR (95% CI)**	**P**
**Change in IGF-1R expression**		0.026		0.086
Unchanged vs downregulation	3.91 (0.92–16.6)	0.064	3.60 (0.84–15.4)	0.084
Upregulation vs downregulation	7.62 (1.64–35.5)	0.010	5.71 (1.20–27.2)	0.028
Upregulation vs unchanged	1.95 (0.89–4.25)	0.094	1.59 (0.71–3.53)	0.26
**Tumor stage**				
III/IV vs I/II	3.52 (1.07–11.6)	0.038	2.71 (0.80–9.13)	0.11

**Fig 3:**
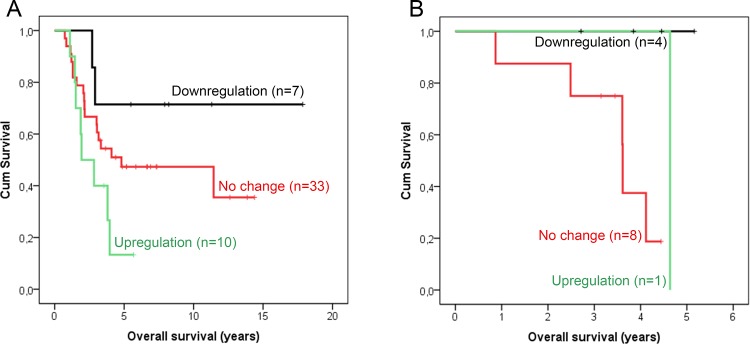
Kaplan-Meier survival curves. Kaplan-Meier survival curve estimates of overall survival of chemotherapy (**A**) or endocrine therapy (**B**) treated patients with a tumor showing a downregulation, no change or an upregulation in IGF-1R expression after neoadjuvant treatment (p = 0.060 and p = 0.069, respectively).

Since membranous IGF-1R expression changed during neoadjuvant treatment, we analyzed whether these changes were associated with OS. Kaplan-Meier analysis showed a significant association (log-rank test, p = 0.013, Fig. 2B). Patients whose tumors showed an increase in membranous IGF-1R expression after neoadjuvant therapy had a significantly shorter mean OS compared to patients with no change in expression or a downregulation. Mean OS time for an upregulation, no change, and a downregulation in membranous IGF-1R expression was 3.0 ± 0.5 years, 7.3 ± 1.0 years and 15.0 ± 1.8 years, respectively. Subgroup analysis for patients with metastatic versus non-metastatic disease is presented in Fig. 2B and 2C. In addition, the chemotherapy and endocrine therapy treated tumors were analyzed separately to determine whether the type of treatment may affect the association between change in IGF-1R and OS (Fig. 3). Although the numbers of patients in these subgroups were small, in both treatment groups the patients with a decrease in IGF-1R expression seem to have the longest overall survival (log-rank test chemotherapy p = 0.060, endocrine therapy p = 0.069).

Since changes in membranous IGF-1R expression were significantly associated with tumor stage, multivariate Cox regression analysis was performed. After correction for tumor stage, patients whose tumors showed an increase in IGF-1R expression still had a poorer prognosis compared to patients with tumors showing a decrease in IGF-1R expression (Table 4).

Changes in cytoplasmic IGF-1R expression and changes in other immunohistochemical markers (ER, PR, HER2) were not significantly associated with survival. Ki67 was significantly reduced after neoadjuvant treatment (paired t-test, p = 0.008). However, Ki67 expression itself, or changes in expression, were not significantly associated with IGF-1R expression, changes in IGF-1R expression, and survival. Cleaved caspase-3 expression did not differ significantly before and after neoadjuvant treatment and was not associated with IGF-1R expression, changes in IGF-1R expression, and survival.

## Discussion

To our knowledge, this is the first study which showed that changes in membranous IGF-1R expression during neoadjuvant breast cancer treatment are significantly associated with OS. Patients with tumors that showed an upregulation in IGF-1R expression had a shorter OS compared to patients with a down-regulation in IGF-1R expression. In addition, we found that baseline membranous IGF-1R expression was significantly associated with hormone receptor positivity, lower tumor stage and longer OS, which is in line with previous research.[6,7,21,22]

In patients receiving endocrine treatment, a down-regulation of membranous IGF-1R was observed more frequently than an upregulation. Although this subgroup consisted of only 13 patients, this is in line with previous reports.[14] Recently, we have shown that a pre-operative short course exposure to tamoxifen or anastrazole can induce a downregulation in IGF-1R expression in patients with primary hormone receptor positive breast cancer.[12] This down-regulation in IGF-1R may reflect response to endocrine therapy, since IGF-1R expression is regulated by estrogen, which can potentially explain the prolonged survival in these patients. In another study, the expression of IGF-1R was analyzed in patients with recurrent breast tumors treated with tamoxifen for at least 6 months. IGF-1R expression was significantly lower in the recurrent lesions, compared to the primary tumors.[14] These results show that endocrine therapy can downregulate IGF-1R expression and, therefore, this result argues against combining endocrine therapy with IGF-1R inhibitors, or treating hormone therapy resistant breast tumors with anti-IGF-1R antibodies.

A few previous clinical studies have shown changes in IGF-1R expression in breast cancer after endocrine therapy.[14] However, chemotherapy induced changes in IGF-1R in breast cancer have not been reported before. This study showed that chemotherapy can induce both an upregulation and a downregulation of membranous IGF-1R expression. Most importantly, our data demonstrate that changes in membranous IGF-1R expression may be a prognostic factor in neoadjuvantly treated breast cancer patients. Patients with tumors showing an increase in membranous IGF-1R expression had a shorter overall survival, compared with patients with tumors showing a down regulation in IGF-1R expression. The observation that treatment induced upregulation is associated with poor outcome may suggest that IGF-1R plays a role in treatment resistance. There are several ways by which IGF-1R can play a role in therapy resistance. For example, IGF-1R may protect cancer cells from apoptosis by activation of the PI-3K/AKT pathway or protect cells from drug-induced cytostatic effects by activation of the MAPK pathway.[23] Moreover, Gou ea. stated that IGF-1R can promote multidrug resistance (MDR) by increasing the expression of MDR-related genes such as mdr-1, c-H-ras and MnSOD.[24]

Although upregulation of membranous IGF-1R expression seems to be a poor prognostic factor, patients with these tumors may potentially benefit from IGF-1R targeted therapy. Phase I/II studies with these agents have shown the safety and tolerability of targeting IGF-1R and have demonstrated a wide range of responses, from progressive disease to near complete response.[25–28] However, larger randomized phase II//III trials failed to show a clear benefit from targeting IGF-1R in combination with conventional treatment strategies.[29–31] It should be noted that these trials were conducted in large unselected patient populations, while studies have shown that IGF-1R expression is a prerequisite for antitumor activity of anti-IGF-1R antibodies.[32–34] Thus, patient selection for IGF-1R targeted therapy could be based on receptor expression and patients showing increased IGF-1R expression during conventional anti-cancer treatment may potentially benefit from IGF-1R targeted treatment. Of note, the cellular location of IGF-1R (membranous versus cytoplasmic) seems to be of importance, since membranous expression was a strong prognostic factor, whereas cytoplasmic expression was not. Moreover, Asmane et al. reported that exclusive intranuclear IGF-1R staining was associated with progression free survival of sarcoma patients who were treated with an anti-IGF-1R antibody.[34] However, exclusive nuclear staining of IGF-1R was not observed in this study.

We attributed the changes in IGF-1R expression to neoadjuvant treatment. However, other potential explanations need to be addressed. First, staining was performed on archival tumor tissue. Pretreatment expression was analyzed on a single biopsy and post treatment expression was analyzed using one representative tumor section from the resection material. Due to possible heterogeneous IGF-1R expression within the tumor, sampling errors may have occurred. Second, tissue processing for biopsies and surgical resection material were different. Parameters such as time between excision and fixation, duration of fixation, and type of fixative can potentially affect the immunoreactivity of the antigen and thus the immunohistochemical staining. To our knowledge, no information is available on the effect of tissue fixation on IGF-1R staining. However, several studies have shown that the concordance rates for comparable markers like ER, PR, HER2 and Ki67 are high between biopsies and surgical resection material.[35–37] Third, differences in IGF-1R expression may occur naturally during the course of disease, for example due to disease progression. Fourth, our sample size was relatively small and thus findings may have occurred by coincidence. And finally, this study was performed only for neoadjuvant treated breast tumors and it contained a high number of HER2-overexpressing tumors. Of all biopsies, 43% overexpressed HER2 (immunohistochemistry score 3+) while literature reports that only 15 to 30% of the newly diagnosed breast tumors overexpress HER2.[38] Also, it is unknown whether our results can be extrapolated to other settings, such as adjuvant breast cancer treatment. The precise role of these factors in our patient population cannot be assessed since this was a retrospective non-randomized study. Therefore, future prospective studies are warranted to confirm our findings.

## Conclusion

Neoadjuvant breast cancer therapy can induce changes in IGF-1R expression. An upregulation of membranous IGF-1R expression after neoadjuvant treatment is a poor prognostic factor in breast cancer patients, providing the rationale for incorporating anti-IGF-1R drugs in the management of this patient group. Future studies in larger patient populations are warranted to confirm these findings.
